# Model-Base Estimation of Non-Invasive Ventilation Weaning of Preterm Infants Exposed to Osteopathic Manipulative Treatment: A Propensity-Score-Matched Cohort Study

**DOI:** 10.3390/healthcare10122379

**Published:** 2022-11-27

**Authors:** Andrea Gianmaria Tarantino, Luca Vismara, Francesca Buffone, Giuliana Bianchi, Andrea Bergna, Monica Vanoni, Claudia Tabbi, Ilia Bresesti, Massimo Agosti

**Affiliations:** 1 Division of Paediatric, Manima Non-Profit Organization Social Assistance and Healthcare, 20125 Milan, Italy; 2 Department of Research, Institute Osteopathy Milan, Istituto Osteopatia Milano (SOMA), 20126 Milan, Italy; 3 Division of Neurology and Neurorehabilitation, IRCCS Istituto Auxologico Italiano, 28824 Piancavallo-Verbania, Italy; 4 Principles and Practice of Clinical Research (PPCR), Harvard T.H. Chan School of Public Health–ECPE, Boston, MA 02115, USA; 5 Woman and Child Department, Varese Hospital, Insubria University, Via Ravasi 2, 21100 Varese, Italy

**Keywords:** osteopathic manipulative treatment, preterm infants, non-invasive ventilation weaning, neonatal intensive care unit

## Abstract

Ventilation weaning is a key intensive care event influencing preterm infants’ discharge from a neonatal intensive care unit (NICU). Osteopathic manipulative treatment (OMT) has been recently introduced in some Italian NICUs. This retrospective cohort study tested if OMT is associated with faster non-invasive ventilation (NIV) weaning. The time to NIV weaning was assessed in very preterm and very low birth weight infants who either received or did not receive OMT. The propensity score model included gender, antenatal steroids, gestational age (GA), birth weight (BW), and Apgar score 5′. Out of 93 infants, 40 were included in the multilevel survival analysis, showing a reduction of time to NIV weaning for GA (HR: 2.58, 95%CI: 3.91 to 1.71, *p* < 0.001) and OMT (HR: 3.62, 95%CI: 8.13 to 1.61, *p* = 0.002). Time to independent ventilation (TIV) was modeled with GA and BW as dependent variables and OMT as the factor. A negative linear effect of GA and BW on TIV was shown. OMT exposure studied as the factor of GA had effects on TIV in infants born up to the 32nd gestational week. Preterm infants exposed to OMT were associated with earlier achievement of NIV weaning. This result, together with the demonstrated OMT safety, suggests the conduct of clinical trials in preterm infants younger than 32 weeks of GA.

## 1. Introduction

Very preterm infants often need positive pressure ventilatory support due to the immaturity of their chest wall pulmonary system; the reduced quantity and quality of surfactant; the neurological and thoracoabdominal asynchrony; the weakness of the intercostal muscles; and the relative shape and position of the diaphragm affecting ventilation, causing atelectasis and subsequently the tendency of the lungs to collapse. To prevent respiratory failure, respiratory distress syndrome (RDS), and bronchopulmonary dysplasia (BPD), preterm infants often require the support of non-invasive ventilation (NIV) [1,2,3,4,5,6,7].

However, even though respiratory support is fundamental during intensive care, recent studies showed that a too-long period—either of invasive ventilation or non-invasive ventilation—is associated with an increased risk of mortality or neurodevelopmental disability, due to the hemodynamic instability and cerebral inflammatory response [8,9]. Therefore, any therapy combined with NIV that can enhance the infants’ adaptation and cardiorespiratory function—consequently reducing the time of ventilation—would have a significant impact on morbidity and mortality. Moreover, since the accomplishment of an autonomous and stable cardiorespiratory function is one of the criteria for discharge [10,11], it would also have a positive influence on human costs and expenses of the health system (only for extremely preterm infants is there a mean cost of USD 65,600) [12,13,14,15,16,17].

The osteopathic manipulative treatment (OMT) is a non-invasive complementary manual therapy that enhances the functional integrity of the body, improves the physiological function, and supports homeostasis [18,19]; it evaluates and treats the somatic dysfunction (SD), which is defined as “an impaired or altered function of related components of the body framework system: skeletal, arthrodial, and myofascial structures, and their related vascular, lymphatic, and neural elements” [20,21]. Different studies have evaluated its possible contributions in the pediatric field, looking especially at prematurity: for instance, OMT is related to reduced timing to oral feeding, shorter LOS, and improvements in cardiac frequency and oxygen saturation [22,23,24,25,26,27,28]. Moreover, a recent study described the correlation of SD severity assessed with the Variability Model and the vagal tone in preterm infants [29]. In this perspective, the OMT added to the standard therapies may play a fundamental role in the short-term effects during the stay in the neonatal intensive care unit (NICU) and in the medium- and long-term effects for the prevention of respiratory diseases.

The main aim of this propensity-score-matched retrospective cohort study is to understand if the OMT is associated with faster NIV weaning; as secondary aims, it was assessed if the OMT is also associated with LOS, anthropometric growth, and time to autonomous respiration.

## 2. Materials and Methods

This is a single-center propensity-score-matched retrospective cohort study: the data were collected from the tertiary level NICU of Del Ponte Hospital in Varese from March 2015 to January 2017. All the peri- and post-natal data—including information about demographic entities, delivery, medical procedures, and perinatal and maternal information—were retrieved from an electronic dataset. During the hospital stay, a multidisciplinary team evaluated the infants; it was composed of neonatologists, pediatric nurses, physical therapists, and pediatric osteopaths. An expert neonatologist was available for immediate consultation during OMTs. The Provincial Ethics Committee of Varese approved the study on 02/14/2017. The manuscript was edited in conformity with STROBE guidelines.

### 2.1. Patients

The study population included very preterm and very low birth weight (VLBW) infants—i.e., with gestational age (GA) between 26 and 32 weeks and birth weight (BW) < 2000 g—treated with NIV support; they could be a singleton or twins. Infants affected by major congenital malformations, severe neurological disease (i.e., intraventricular hemorrhage, cerebral palsy), severe cardiovascular diseases, triplet births, or infants treated with intubation were not screened for eligibility. A total of 40 out of 94 infants were fully eligible and were propensity score matched on the basis of relevant demographic and clinical perinatal parameters.

### 2.2. Non-Invasive Ventilation Strategy

NIV was performed on the basis of the NICU guidelines for assisted ventilation, that is,

- when infants were at risk of RDS;
- when infants with a GA < 30 weeks did not need mechanical ventilation;
- when FiO2 > 0.30 from nasal cannula or mask to keep oxygen saturation;
- when PaO2 < 0.50 mmHg or in the case of apnea of prematurity.

A loading dose of intravenous caffeine (20 mg/Kg) was administered at NICU admission, followed by a maintenance dose of 5–10 mg/Kg. The FiO2 was adjusted by a physician to stabilize SaO2 at 90–95%. All the ventilatory support was delivered by CNO (Medin, Olching, Germany).

### 2.3. Weaning Strategy

The routine clinical management depended on the infants’ conditions; hence, it was adjusted day by day following the oxygen requirement. The weaning started after 24 h of stable ventilatory parameters at a pressure of 4–5 cmH2O. The protocol of weaning in the ward was a gradual weaning at “ON–OFF” intervals. It started with an “OFF” period, during which the infant stayed in the crib oxygen or air as long as the ventilatory and cardiovascular parameters were stable. Then, there was a fixed “ON” period of 6 h. During these “ON–OFF” intervals, it was expected that we would see a gradual incremental pattern of the “OFF” periods. The “ON–OFF” interval ended when the infant was stable for at least 16 h during the “OFF” period, whereas if the baby met a failure criterion, a 6-h “ON” period was recommended.

Failure criterion considered at least 2 of the following points:

- Increased breathing load (use secondary and accessory muscles), with a respiratory rate > 75 bpm.
- Increased apneas or bradycardia event based on the previous 6 h (>2 in 1 h).
- pH < 7.2.
- Increased oxygen requirement to maintain saturation 86% or PaO2 < 45mmHg.
- PaCO2 > 65 mmHg.
- Major events requiring resuscitation.

### 2.4. Exposure Variable

The OMT was the exposure variable; it was performed by trained pediatric osteopaths with more than 3 years of experience in the NICU. OMTs were performed after 1–5 days from birth, twice a week until discharge for a duration of up to 30 min per infant. Our NICU used a standardized protocol for safety during treatment sessions and it was based on SaO2 > 0.85, more than 3 apneas, or alarming heart rate (<100 or >150 bps). If one of these scenarios occurred, the session was suspended or not performed.

Osteopaths were trained to perform a specific examination protocol using the variability model of SD [21,29]. The exam was composed as follows:

1. The osteopath with a manual palpatory grip between the skull and the sacrum evaluated the quality of movement and mobility on the three planes of movement. The examiner assessed the movement variability within the neutral zone (NZ) of the spine with a sacro-occipital stance [29]. The examiner checked the motion reaction of the spine fascia with a light reverse rotary, opposite lateral, and traction and compression stimulus (micromotion), induced between the head and the sacral regions. If the multiplanar movements were symmetrical, the newborn was considered functional, and vice versa if the movement inside the NZ was asymmetrical, wherein the newborn was considered dysfunctional. If there was a SD, osteopaths continued with evaluation.
2. Myo-fascial tissue texture (TT) assessment was performed using a bimanual compression in the scapular, pelvic girdle, lower chest, upper chest, diaphragm, and abdominal and spinal regions, as well as using a gentle compression of the fascia of the spine with the same sacro-occipital stance. An altered elastic response to compression defined an atypical tissue resilience and consequently an abnormal TT.
3. Finally, to evaluate the tenderness status, the reaction during slight compression between the head and sacrum was evaluated using facial expression, the presence of reflex moves, crying, or the sudden increase in heart rate.

The examiner assessing the variability of movement within the NZ was the fundamental analysis guiding all other assessments. At the end of the procedure, two OMT practitioners assigned the SD severity to the infant, considering the variability of movement, TT, and tenderness. Consequently, the manipulative treatment was decided on the basis of the SD grade. The principal guide to osteopathic anamnesis has always been the alteration of motion symmetry in NZ. Osteopathic treatment of SD used cranial, visceral, and myofascial release techniques. The most dysfunctional areas were the skull base, the diaphragmatic area, and the dorsal and costal area.

### 2.5. Follow-Up

The multidisciplinary team observed the patients and recorded the clinical events on daily bases for medical, nurse, and ventilatory support data. All the information was collected in an accessible hospital database, from which we performed the data extraction and analysis.

### 2.6. Outcomes

The time for ventilation weaning was considered as the time in days that occurred from the NIV initiation to the full independence from ventilation. Hence, in our statistical model, the event was the ventilation weaning, and the time variable was the time to independent ventilation (TIV), as previously defined. Other assessed perinatal and clinical variables were gender; antenatal steroids (ANS); GA; BW; body length; head circumference; Apgar at 1 and 5 min from birth; the number of small for gestational age infants (SGA); the type of NIV; and the occurrence of apneas, BPD, RDS, or patent ductus arteriosus (PDA).

### 2.7. Statistical Analysis

Clinical and demographic variables were reported with descriptive statistics for continuous variables (mean and standard deviation) and with frequencies for categorical variables (percentages). To deal with sampling bias and potential confounders, a propensity score model was implemented using a model including gender, ANS, GA, BW, and Apgar score 5′. The nearest-neighborhood matching method with a 1:1 ratio was used, applying a caliper of 0.1 of the standard deviation of the logit of the propensity score. Distribution and normality were tested with formal tests, whereas the potential imbalance after matching was tested at baseline between groups using the chi-squared test for categorical variables and the two-sided Student’s t-test for continuous variables.

For the primary outcome, patients were observed until ventilation weaning. The effect of OMT exposure compared to the control group was graphically depicted with a Kaplan–Meier curve, including a risk table, and formally tested with a Log-rank test. The multivariable Cox proportional hazard model was used to compare the time to event in exposed and non-exposed groups, both adjusted for potential covariates. The proportional hazard assumption was tested with a phtest that did not reject the null hypothesis that the hazards were proportional to our model. To study the secondary endpoints, we used multivariate linear models for the prediction of TIV in different GAs and BWs and considered OMT as the factorial variable. For simplicity, the models were graphically reported. Statistical significance was considered if the *p*-value was <0.05. Statistical analyses were performed with STATA version 15 and R software version 4.5, as needed.

## 3. Results

Out of 94 screened infants, 20 infants per group were eligible and were included via the propensity score matching. At baseline, there was homogeneity for the main variables in the two groups, and the propensity score efficacy was tested with the standardized mean difference (SMD). Concerning the diseases, both groups mainly reported RDS, followed by PDA, apneas, and BPD. Instead, the prevalent type of NIV was nasal high-frequency oscillatory ventilation (NHFOV), followed by NCPAP. On average, a TIV difference of 9.85 (−0.93 to 20.62) days was registered between groups combined with a difference of 5.34 (−10.07 to 21.98) days of LOS, which was lower in the exposed group, although non-statistically significant (see Table 1).

**Figure 1:**
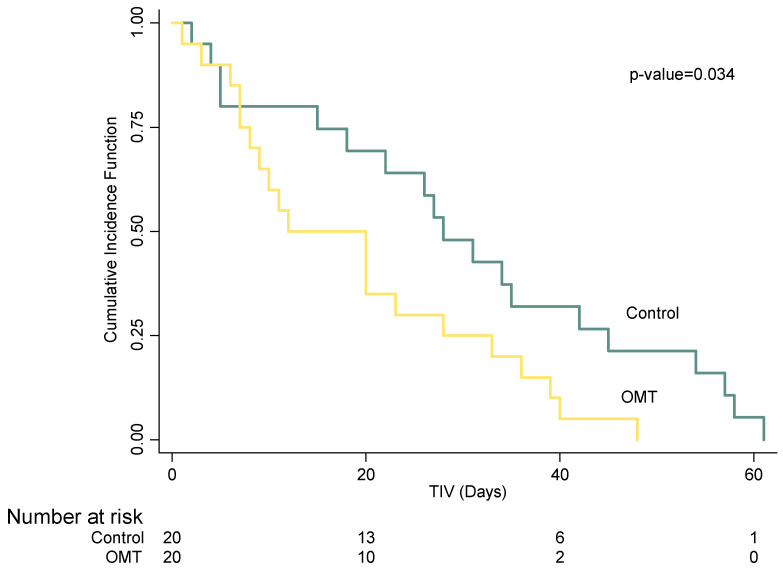
Distribution of cumulative incidence function of NIV weaning in infants stratified by OMT exposure.

**Figure 2:**
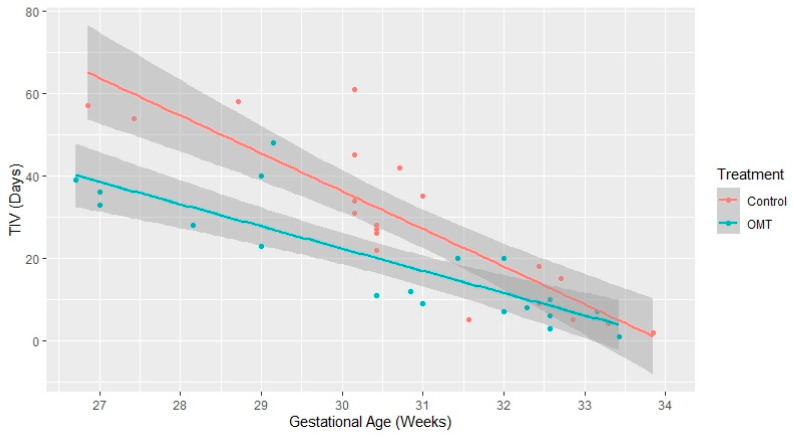
Distribution of ventilation weaning in the function of the gestational age stratified by OMT exposure.

**Figure 3:**
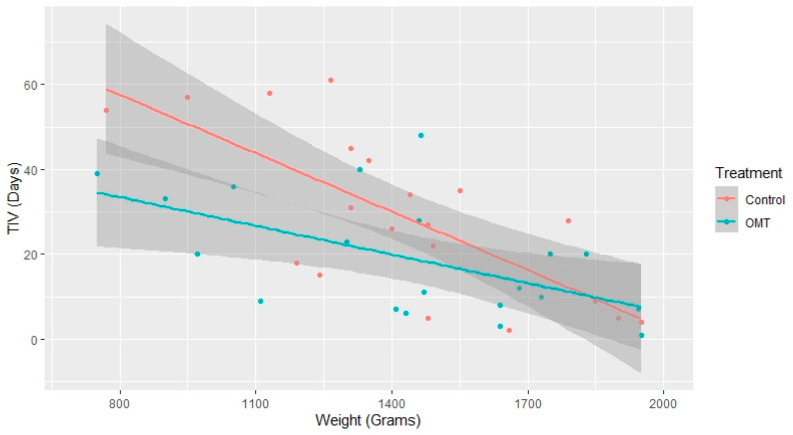
Distribution of ventilation weaning in the function of the birth weight stratified by OMT exposure.

All the observed infants reached NIV weaning. The univariate survival analysis shows that the OMT group had a statistically significantly lower time-to-event (*p* = 0.034), as depicted in Figure 1. As reported in Table 2, after adjusting for potential covariates (i.e., PDA, BPD, RDS, gender, Apgar score 5′, VLBW, apnea of prematurity, GA, and OMT), the multivariate analysis showed a statistically significant difference for GA (HR: 2.58, 95% CI: 3.91 to 1.71, *p* < 0.001) and OMT (HR: 3.62, 95% CI: 8.13 to 1.61, *p* = 0.002). The multivariate linear model analyzed the relationship between TIV and GA, as well as TIV and BW in both the exposed and non-exposed groups. Figure 2 reports the mean total and 95% boundary of TIV measured in days and GA measured in weeks: it shows a negative linear effect of GA on TIV, with the OMT exposure that significantly shortened the time to ventilation weaning in infants born before the 32nd week of GA. Instead, Figure 3 reports the mean total and 95% boundary of TIV measured in days and BW measured in grams, showing a negative linear effect of BW on TIV with no significant differences between groups.

The exposed group received a total of 299 OMT sessions. Out of 299 of these sessions, 2 of them (0.66%) were interrupted according to the previously mentioned safety protocol, without reporting any permanent mild adverse event or medical staff intervention. There was an average of 14.5 treatments per infant during the NICU permanence with a mean of three manipulative techniques performed within each treatment session. The diaphragmatic region was the most frequently treated, showing a mean relative frequency of treatment of 0.95, followed by the abdominal region (0.62) and craniosacral techniques (0.51). More details on the treatment descriptive analysis are reported in Table 3. From a qualitative point of view, despite both exposed and non-exposed infants presenting compensatory prolonged ventilation during the “OFF” period, it occurred more frequently in the exposed group.

## 4. Discussion

This model-based retrospective cohort study described the weaning time from NIV in preterm infants either exposed or not exposed to OMT during NICU permanence. The study protocol included propensity score matching to reduce the presence of confounders during analysis. As the primary endpoint, the analysis included the time-to-event assessment performed with survival analysis. Our multilevel model shows that the increase in GA (HR: 2.58) and the OMT exposure (HR: 3.62) enhanced the weaning probability. This result agrees with a review on continuous positive airway pressure (CPAP) weaning, highlighting the direct relationship between weaning and GA [30]. Concerning TIV, when the infants were more mature, they achieved weaning with a similar time in both groups; however, premature infants with GA < 32 weeks exposed to OMT reached autonomous ventilation more quickly (see Figure 2). Lastly, in our multivariate Cox regression model and the multilevel linear regression model stratified per OMT exposure, infants’ BW seems to have had no interaction with TIV (see Figure 3).

In the last twenty years, study groups have improved the analysis of term and preterm infants’ breathing patterns in therapy with NIV [30,31,32,33,34]. Due to the immaturity of the lung system, very preterm and extremely preterm infants frequently need ventilatory support. NIV has been increasingly used as a ventilatory strategy because it prevents chronic lung diseases, thanks to the reduced traumatic impact on lung structures and the less invasive oxidative stress [30,31,32,33,34,35,36]. Nonetheless, NIV management and weaning often fail due to the immaturity of the preterm infants’ chest wall pulmonary system [37,38]: the unbalance between pulmonary and chest wall compliance leads to inefficient pulmonary insufflation during diaphragmatic contraction [7]. Therefore, due to the inefficient negative pressure in the pleural space, the alveoli remain collapsed, reducing the exchange space of ventilation [8]. The preterm infants described herein frequently presented retractions in the diaphragmatic region and the abdominal wall (right iliac fossa), thus confirming this dysfunctional pattern. When the chest wall is not sufficiently rigid and the diaphragmatic/abdominal muscles are not balanced and efficient, premature babies may experience difficulties to maintain a functional inflation pressure during the “OFF” periods, thus increasing the risk of weaning failure. Another observed pattern was the use of compensatory respiration, characterized by a deep inspiration followed by a prolonged expiration. This is a well-known strategy adopted by preterm infants and is supposed to compensate for increased chest wall compliance, enhancing pulmonary inflation [39]. Compensatory respiration involves glottal movements, which require the competence of larynx muscles: a laryngeal muscle contraction produces prolonged expiration; this helps to maintain the increased pulmonary pressure during expiration [40]. We noticed that during the “OFF” period, babies exposed to OMT more often used deep breaths followed by prolonged expiration periods.

Speculatively, the OMT may influence the somatosensory system, thus causing differences between groups. Myelinations of pre- and post-central gyri proceed actively around the 35th gestational week [41]. Therefore, the interhemispheric connectivity of the somatosensory and motor cortex exponentially increases during the preterm period [42], which plays a critical role in newborns’ resting-state neural activity. While the adults’ brain has the cortical hubs of resting-state networks in higher-order association areas (medial/lateral prefrontal cortices, lateral parietal, lateral temporal, and posterior cingulate) [43], the newborns’ brain has the cortical hubs in the somatosensory and motor regions with interaction with talamo-cortical networks [44]. This suggests that treating the tissue rigidity and myofascial strain may influence the bottom-up somatosensory modulation during a crucial period for central sensorimotor pairing.

Both observed patterns may explain the increased ratio of NIV weaning by time in the exposed group and describe a significant oxygenation increment in preterm infants after an OMT session [29].

This study has limitations. First, the information on drug administration during NICU permanence is limited by database details. Despite the propensity score matching, the therapeutic protocols may have slightly differed due to the physician’s assignment. This potential imbalance between groups may have led to a bias toward the null hypothesis in our results. Secondly, there may have been treatment heterogeneity due to the intrinsic modality of administration of manual techniques (such as OMT), case, experience, and manual abilities with consequences on efficacy and safety. The osteopaths who provided the treatment were experienced and trained for the NICU setting, thus leading to issues with the generalizability of our results. Finally, despite the promising trend, due to the retrospective nature of this study, the database did not contain detailed information about ventilatory and cardiovascular parameters within the OMT context; hence, it is not possible to draw conclusions on OMT biological effects in this cohort.

Our study has introduced a new research question about OMT efficacy on TIV reduction, as well as its possible biological implications. Therefore, adequately powered RCTs should assess the BPD incidence and rate of NIV failure in infants with a GA < 32 weeks treated with OMT to evaluate its effects. Moreover, the results of this study show the safety and feasibility of OMT even at early stages, as well as its positive influence on preterm infants’ respiratory adaptation, thus suggesting plausible effects on national healthcare service.

## Figures and Tables

**Table 1:** Demographic and clinical characteristics of the included infants stratified by exposition to OMT. The balance between groups was tested with Student’s t-test and the chi-squared test as appropriate. Propensity score efficacy was tested with the standardized mean difference (SMD). Efficacy on balance between variables was set at SMD < 0.10.

Variable	Unexposed Group (N = 20)	Exposed Group (N = 20)	*p*-Value	SMD
**Male**–n. (%)	10 (50)	11 (55)	0.752	−0.110
**Perinatal characteristics**				
ANS—n. (%)	8 (40)	8 (40)	0.999	0
Body weight—g.	1425.2 ± 306	1435.5 ± 356.6	0.922	0.031
Apgar 5 min—pt.	8.15 ± 1.22	8.10 ± 1.37	0.903	−0.039
Head circumference—cm.	28 ± 2.7	28.9 ± 2.3	0.868	0.359
Gestational age—wk.	31.5 ± 1.63	31.6 ± 1.64	0.763	0.061
SGA—n. (%)	7 (35)	8 (40)	0.744	0.117
ELBW—n. (%)	2 (10)	3 (15)	0.633	0.255
LBW—n. (%)	4 (20)	5 (25)	0.585	0.156
Body length—cm.	39.1 ± 3.7	39.9 ± 3.71	0.520	0.216
VLBW—n. (%)	14 (70)	12 (60)	0.507	0.243
**Clinical characteristics**				
RDS—n. (%)	12 (60)	12 (60)	0.999	0
Apnea—n. (%)	9 (40)	8 (45)	0.749	0.119
PDA—n. (%)	9 (45)	11 (55)	0.527	0.221
BPD—n. (%)	8 (40)	6 (30)	0.507	−0.243
TIV—days	28.90 ± 4.30	19.05 ± 3.12	0.072	−2.622
**Type of ventilation**				
NHFOV—n. (%)	12 (60)	12 (60)	-	0
NCPAP—n. (%)	5 (25)	4 (20)	-	−0.158
BiPAP—n- (%)	3 (15)	4 (20)	0.881	0.192
**Propensity Score—prob.**	0.381 ± 0.214	0.383 ± 0.212	0.969	0.009

**Legend:** antenatal steroids (ANS); low birth weight (LBW); very low birth weight (VLBW); extremely low birth weight (ELBW); small for gestational age (SGA); bronchopulmonary dysplasia (BPD); respiratory distress syndrome (RDS); patent ductus arteriosus (PDA); time of independent ventilation (TIV); nasal high-frequency oscillatory ventilation (NHFOV); nasal constant positive airway pressure (NCPAP); bilevel positive airway pressure (BiPAP).

**Table 2:** Multivariate Cox regression of time of independent ventilation function.

Variable	Hazard Ratio	UCI	LCI	*p*-Value
VLBW	1.024	2.915	0.358	0.966
Apnea	1.176	2.902	0.477	0.724
RDS	0.669	1.685	0.266	0.395
Male	0.673	1.605	0.282	0.373
BPD	0.572	1.293	0.253	0.180
PDA	0.499	1.274	0.195	0.146
Apgar 5′	0.678	1.026	0.447	0.066
OMT	3.625	8.139	1.614	0.002
GA	2.581	3.908	1.705	<0.001

**Legend:** very low birth weight (VLBW); bronchopulmonary dysplasia (BPD); respiratory distress syndrome (RDS); patent ductus arteriosus (PDA); gestational age (GA); osteopathic manipulative treatment (OMT).

**Table 3:** Characteristics of treated regions, number of performed treatments, number of performed manipulative techniques, and relative frequency (RF) of treated region per treatment.

	N	Mean (RF)	SD (RF)	Min (RF)	Max (RF)
NTRT	20	14.9	6.94	7	30
NMT	20	45.3	12.1	20	87
Diaphragm	20	14.4 (0.95)	6.89 (0.13)	6 (0.75)	27 (1)
Abdominal	20	9.32 (0.63)	4.7 (0.14)	3 (0.416)	22 (0.90)
CST	20	7.74 (0.51)	3.67 (0.14)	2 (0.28)	12 (0.85)
Lumbar/pelvic	20	5.45 (0.38)	3.18 (0.18)	0 (0)	15 (0.77)
Cervical	20	3.75 (0.25)	2.04 (0.12)	0 (0)	7 (0.46)
Dorsal	20	1.75 (0.12)	1.65 (0.15)	0 (0)	5 (0.57)

**Legend**: number of treatment sessions (NTRT); number of manipulative techniques (NMT); craniosacral techniques (CST).

## Data Availability

The datasets generated during and/or analyzed during the current study are available in the Zenodo repository with the identifier https://doi.org/10.5281/zenodo.7125136. Accessed on 29 July 2022.
